# Supplementary material for: Monitoring of Inflammatory Bowel Disease in Pregnancy: A Review of the Different Modalities
Source: J Clin Med. 2023 Nov 27;12(23):7343. doi: 10.3390/jcm12237343 (PMC10707304; doi:10.3390/jcm12237343)
Supplement: Supplementary file 1 [file jcm-12-07343-s001.zip › jcm-2661875-supplementary.pdf]

Supplementary Table S1. Intestinal Ultrasound Parameters.

IUS Parameters

1. Bowel wall thickness
    - < 3 mm (0)
    - > 3 mm (1)
2. Bowel wall flow
    - No flow (0)
    - Small circular intramural signal (1)
    - Longer linear intramural signal (2)
    - Longer stretch of vascular signal with extension into mesenteric (3)
3. Bowel wall stratifications
    - Normal echo stratification (0)
    - Focal < 3 cm disruption (1)
    - Extensive >3 cm disruption (2)
4. Small bowel peristalsis
    - Absent (0)
    - Present (1)
5. Mesenteric lymph node
    - Absent (0)
    - Present of LN more than or equal to 4 mm in short axis diameter (1)
6. Mesenteric inflammatory fat
    - Absent (0)
    - Present (1)
7. Colonic haustra coli
    - Absent (0)
    - Present (2)

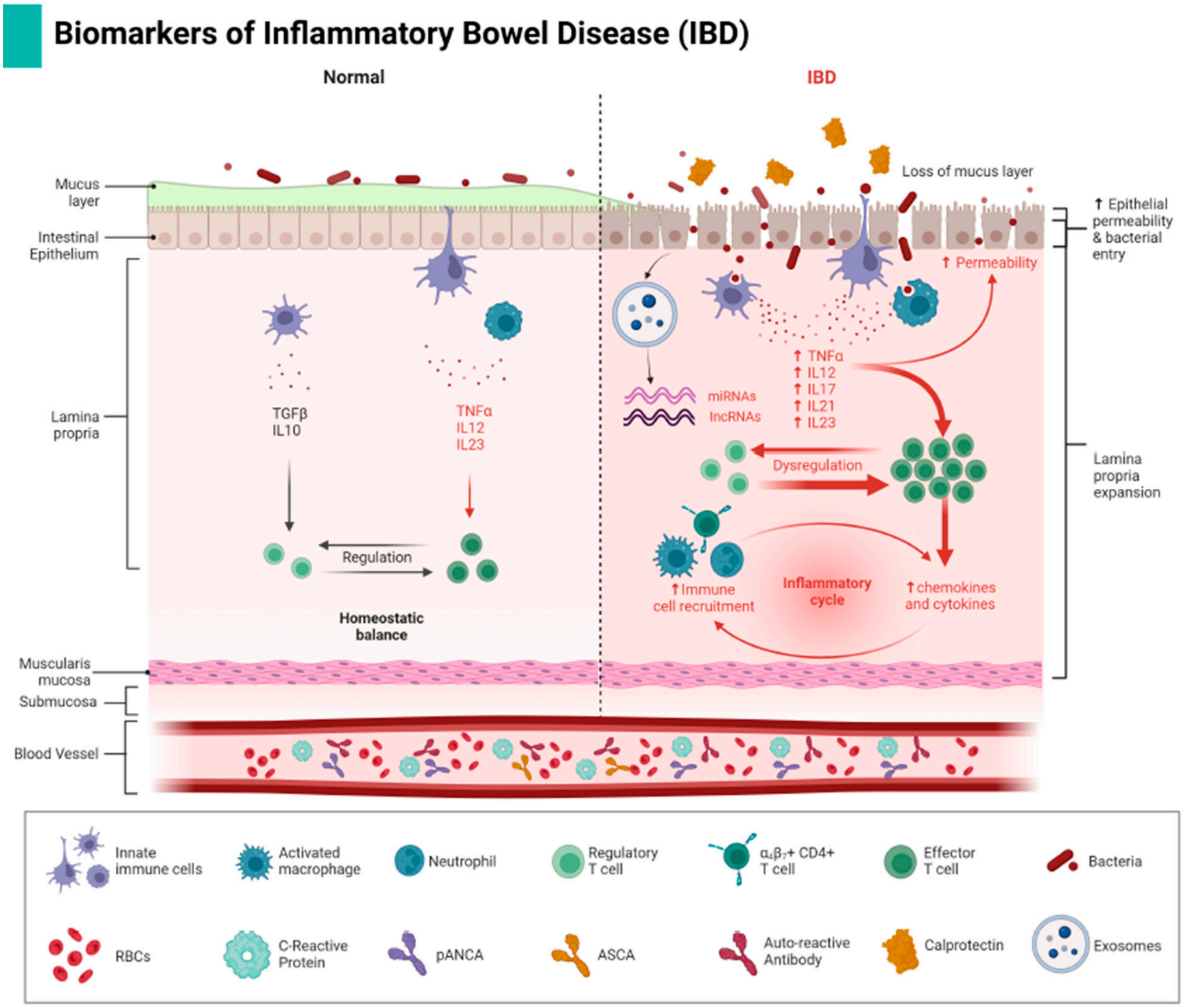

Supplementary Figure S1. The figure present a comparative illustration of intestinal homeostasis and inflammatory Bowel Disease (IBD) pathology, highlighting the biomarkers of IBD. In the normal state, the mucus layers is intact atop the intestinal epithelium, underlain by the lamina propria, muscularis mucosa, submucosa, and blood vessels. Regulatory cytokines such as TGFβ and IL10 maintain immune balance. In contrast, the IBD-affected intestine exhibits a compromised mucus layer, heightened epithelial permeability, and increased bacterial invasion. This disruption leads to an inflammatory response characterized by the upregulation of cytokines and chemokines (TNFα, IL12, IL17, IL21, IL21 and IL23), immune cells recruitment, and lamina propria expansion. CRP, C-reactive Protein; pANCA, perinuclear Anti-Neutrophil Cytoplasmic Antibodies; ASCA, Anti-Saccharomyces cerevisiae Antibodies.
